# Visfatin Mediates Malignant Behaviors through Adipose-Derived Stem Cells Intermediary in Breast Cancer

**DOI:** 10.3390/cancers12010029

**Published:** 2019-12-20

**Authors:** Jyun-Yuan Huang, Yen-Yun Wang, Steven Lo, Ling-Ming Tseng, Dar-Ren Chen, Yi-Chia Wu, Ming-Feng Hou, Shyng-Shiou F. Yuan

**Affiliations:** 1 Translational Research Center, Kaohsiung Medical University Hospital, Kaohsiung Medical University, Kaohsiung 807, Taiwan; 2 Center for Cancer Research, Kaohsiung Medical University, Kaohsiung 807, Taiwan; 3 Department of Medical Research, Kaohsiung Medical University Hospital, Kaohsiung 807, Taiwan; 4 School of Dentistry, College of Dental Medicine, Kaohsiung Medical University Hospital, Kaohsiung 807, Taiwan; 5 College of Medical, Veterinary and Life Sciences, University of Glasgow, Glasgow G12 8QQ, UK; 6 Comprehensive Breast Health Center, Department of Surgery, Taipei-Veterans General Hospital, School of Medicine, National Yang-Ming University, Taipei 112, Taiwan; 7 Breast Cancer Center, Changhua Christian Hospital, Changhua 500, Taiwan; 8 Institute of Stem Cell and Translational Cancer Research, Chang Gung Memorial Hospital at Linko, Taoyuan 333, Taiwan; 9 Research Center of Regenerative Medicine and Cell Therapy, Kaohsiung Medical University, Kaohsiung 807, Taiwan; 10 Department of Surgery, Kaohsiung Medical University Hospital, Kaohsiung 807, Taiwan; 11 Center of Teaching and Research, Kaohsiung Municipal Ta-Tung Hospital, Kaohsiung 801, Taiwan; 12 Division of Breast Surgery, Department of Surgery, Center for Cancer Research, Kaohsiung Medical University Chung-Ho Memorial Hospital, Kaohsiung 807, Taiwan; 13 Department of Obstetrics and Gynecology, Kaohsiung Medical University Hospital, Kaohsiung 807, Taiwan; 14 Graduate Institute of Medicine, College of Medicine, Kaohsiung Medical University, Kaohsiung 807, Taiwan; 15 Department of Biological Science and Technology, College of Biological Science and Technology, National ChiaoTung University, Hsinchu 300, Taiwan; 16 Center for Intelligent Drug Systems and Smart Bio-devices (IDS2B), National Chiao Tung University, Hsinchu 300, Taiwan

**Keywords:** breast cancer, visfatin, adipose-derived stem cells (ADSCs), GDF15

## Abstract

Adipose-derived stem cells (ADSCs) have been implicated in tumor growth and metastasis in breast cancer. ADSCs exhibit tumor tropism, and are of increasing clinical relevance due to the autologous fat grafting for breast reconstruction. Although we have previously shown that a high level of the adipocytokine visfatin in human breast cancer tissues correlated with tumor progression mediated by cAbl and STAT3, the effects of visfatin in the tumor microenvironment are unclear. To understand how visfatin modulates breast cancer within the tumor-stromal environment, we examined determinants of breast cancer progression using a visfatin-primed ADSCs-tumor co-culture model. ADSCs were isolated from tumor-free adipose tissue adjacent to breast tumors. ADSCs were treated with or without visfatin for 48 h and then collected for co-culture with breast cancer cell line MDA-MB-231 for 72 h in a transwell system. We found that the MDA-MB-231 cells co-cultured with visfatin-treated ADSCs (vADSCs) had higher levels of cell viability, anchorage independent growth, migration, invasion, and tumorsphere formation than that co-cultured with untreated ADSCs (uADSCs). Growth differentiation factor 15 (GDF15) upregulation was found in the co-culture conditioned medium, with GDF15 neutralizing antibody blocking the promoting effect on MDA-MB-231 in co-culture. In addition, a GDF15-induced AKT pathway was found in MDA-MB-231 and treatment with PI3K/AKT inhibitor also reversed the promoting effect. In an orthotopic xenograft mouse model, MDA-MB-231 co-injected with vADSCs formed a larger tumor mass than with uADSCs. Positive correlations were noted between visfatin, GDF15, and phosphor-AKT expressions in human breast cancer specimens. In conclusion, visfatin activated GDF15-AKT pathway mediated via ADSCs to facilitate breast cancer progression.

## 1. Introduction

Breast cancer is one of the commonest cancers worldwide, with 2.09 million cases annually [1]. Obesity is increasingly seen as one of the most important modifiable risk factors in breast cancer, with epidemiological studies indicating that obese, postmenopausal women exhibit both an increased incidence [2] and poorer prognostic outcomes [3]. The breast itself consists of variable amounts of adipocytes (approximately 90%) and epithelial tissue (10%) [4], with adipose tissue no longer considered an inert tissue due to accumulating evidence regarding the interplay between cancer cells and stromal cells in breast cancer. 

Recently, adipose-derived stem cells (ADSCs) have received attention for their role in the biology of breast cancer. ADSCs are increasingly relevant not only due to rising levels of population obesity, but also due to the advent of autologous fat transfer as a method of breast reconstruction after cancer surgery. Of particular concern is the use of ADSC enrichment in fat grafting techniques that are designed to increase percentage fat engraftment [5,6]. ADSCs may influence breast cancer through several distinct pathways. The breast cancer microenvironment may induce the differentiation of ADSCs into carcinoma-associated fibroblasts that promote tumor growth, with more pronounced effects noted in ADSCs from obese patients [7,8]. ADSCs may also produce multiple factors including insulin-like growth factor (IGF), hepatocyte growth factor (HGF), VEGF, IL8, and TGFβ [9,10,11,12,13], and may induce epithelial-mesenchymal transition (EMT), promoting tumor migration and metastases [14,15]. However, at present, it remains unclear how the ADSC tumor-stromal interaction may be further modulated by factors secreted in the tumor microenvironment. 

Obesity-related cytokines, also known as adipocytokines, have been identified as one such factor [16,17]. Adipose tissues emit a variety of adipocytokines that act through autocrine, paracrine and endocrine manners to influence tumor cell proliferation, migration and invasion in breast cancer [18,19,20]. Among adipocytokines, visfatin, also known as nicotinamide phosphoribosyltransferase (NAMPT) or pre-B-cell colony-enhancing factor (PBEF), regulates growth, apoptosis, inflammation, and angiogenesis in mammalian cells [21]. High levels of visfatin in the tumor microenvironment have been shown to be associated with an increased risk of cancer progression and malignant cancer behavior in breast cancer patients [4,22,23]. Intracellular visfatin functions as a rate-limiting enzyme in the biosynthesis of nicotinamide adenine dinucleotide (NAD), whilst extracellular visfatin exhibits enzyme-like activity on extracellular NAD formation as well as cytokine-like activity through a putative receptor-mediated pathway [21]. Both intracellular and extracellular visfatin have been implicated in tumor development [24,25], with promotion of cell growth via a NF-κB/Notch1 pathway in breast cancer cells, and facilitation of metastasis and angiogenesis via PI3K/Akt, MAPK and c-Abl/STAT3 signaling pathways in human endothelial cells, macrophages and breast cancer cells [23,26,27].

Our prior studies showed that a high level of visfatin in human breast cancer tissues correlated with tumor progression mediated by cAbl and STAT3, using breast cancer cell lines. To understand how visfatin modulates breast cancer within the tumor-stromal environment, we examined determinants of breast cancer progression using a unique visfatin primed ADSC tumor co-culture model. This is the first description of an adipocytokine-ADSC-breast cancer co-culture model that more closely mimics the breast tumor microenvironment. Here, we show that visfatin can act via mechanistically pathways distinct from those previously discovered using tumor cell line models in isolation [23]. 

## 2. Results

### 2.1. Visfatin-Treated ADSCs Promoted the Viability, Anchorage Independent Growth, Migration, Invasion, and Tumorsphere Formation of Breast Cancer Cells

**Figure 1:**
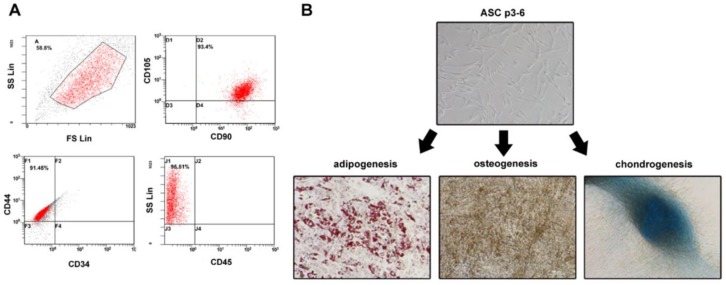
Identification of ADSCs. (**A**) ADSCs were isolated from the adipose tissue of breast tumors. After two to three passages, the expressions of ADSCs markers (CD90FITC, CD105PE, and CD44FITC) and the lack of CD34PE and CD45FITC were confirmed by flow cytometry. (**B**) The differentiation ability of ADSCs was tested by adipogenesis, osteogenesis, and chondrogenesis.

Previous study showed that visfatin enhanced the metastasis of breast cancer cells [22,23] Here, we investigated whether visfatin stimulates ADSCs to promote the breast cancer progression. Using ADSCs isolated from adipose tissues adjacent to breast tumors of breast cancer patients, we were able to demonstrate that the isolated ADSCs showed plastic adherence, expressed mesenchymal and stemness markers and displayed multipotency in both flow cytometry (positive for CD90, CD105, and CD44, and negative for CD34 and CD45) and differentiation assays for adipogenesis, osteogenesis, and chondrogenesis (Figure 1). 

**Figure 2:**
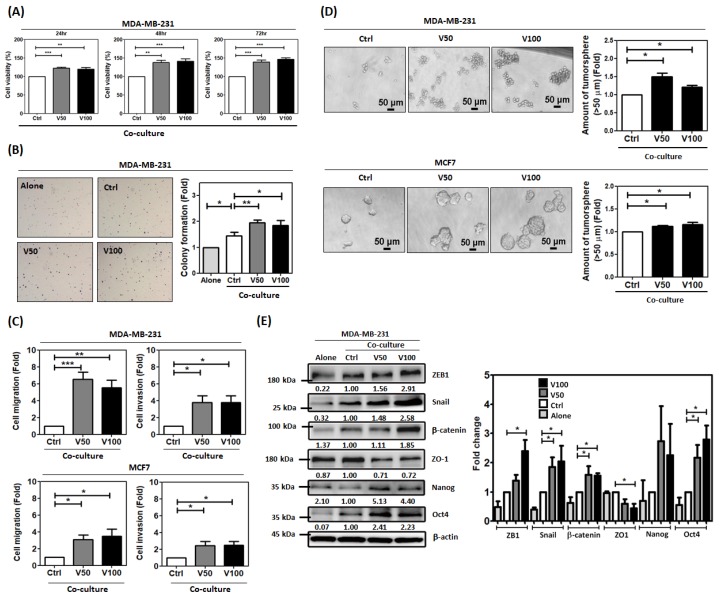
Visfatin-primed ADSCs (vADSCs) promoted the viability, anchorage independent growth, migration, invasion, epithelial-mesenchymal transition (EMT), and stemness property of breast cancer cells. ADSCs were treated with or without visfatin, noted as vADSCs or uADSCs, respectively, at 50 and 100 ng/mL for 48 h. Then, MDA-MB-231 cells were indirectly co-cultured with uADSCs or vADSCs in a transwell system for 72 h, noted as Ctrl or V50 and V100. (**A**) After that, the MDA-MB-231 collected from the co-culture were seeded in 96-well plate for 24, 48, and 72 h for analyzing the cell viability by XTT assay. (**B**) The collected MDA-MB-231 cells were seeded in a six-well plate with Noble agar for 21 days to analyze the anchorage independent growth by soft agar colony formation assay. (**C**) The collected MDA-MB-231 cells were seeded in a 24-well transwell plate coated with or without the Matrigel for performing migration or invasion assay, respectively. (**D**) The collected MDA-MB-231 cells were seeded in a 96-well low binding plate for tumorsphere formation. (**E**) The collected MDA-MB-231 cells were analyzed for the expressions of EMT- and stemness-related proteins by western blotting. MDA-MB-231 cells only were noted as Alone. Representative images of western blot shown. The result was quantified and present as histogram. All experiments were performed in triplicate. The statistical differences were calculated by t-test from three independent experiments. *p*-values < 0.05 or < 0.01 were marked with “*” or “**”, respectively.

The ADSCs with 3–6 passages underwent pre-treatment with visfatin at 50 or 100 ng/mL, which was similar to the medium or high level of breast cancer patients, respectively [23], for 48 h. Then, MDA-MB-231 cells were co-cultured with the visfatin-pretreated ADSCs (vADSCs) or untreated ADSCs (uADSCs) indirectly in a transwell system for 72 h. ADSCs were obtained from 10 breast cancer patients, with visfatin-treatment having a significant promoting effect on MDA-MB-231 cell migration (Figure S1). We chose three ADSCs which promoted greater than 1.5-fold cell migration of MDA-MB-231 to evaluate their effects on the viability, anchorage independent growth, migration and invasion of MDA-MB-231. We found that co-culture with vADSCs promoted the cell viability of MDA-MB-231 at different time points compared to uADSCs (Figure 2A). Also, vADSCs stimulated greater colony formation of MDA-MB-231 compared to uADSCs (Figure 2B). Meanwhile, the cell migration, invasion, and tumorsphere formation of MDA-MB-231 or MCF7 were enhanced after co-culturing with vADSCs compared with co-culturing with uADSCs (Figure 2C,D). Since the process of epithelial-mesenchymal transition (EMT) leads to acquisition of a migratory and invasive phenotype in cancer cells, the expression of EMT-related proteins of MDA-MB-231 co-culture were analyzed. We found that the mesenchymal markers of ZEB, Snail, and β-catenin were increased and the epithelial marker ZO-1 was decreased in MDA-MB-231 cells co-cultured with vADSCs compared to uADSCs (Figure 2E). Since tumorsphere formation is associated with cancer stemness, the expressions of stemness-related proteins were also assessed. We found that the expression of Nanog and Oct4 were upregulated when MDA-MB-231 cells were co-cultured with vADSCs compared to uADSCs (Figure 2E). To clarify the effect of the breast cancer microenvironment on neighboring ADSCs, we tested the response of the ADSCs isolated from non-cancer patients who underwent cosmetic breast surgery to visfatin treatment. The results showed that tumor-free ADSCs did not respond to visfatin treatment to enhance the cell migration of MDA-MB-231 (Figure S2). This suggested that the breast cancer microenvironment may alter the response of ADSCs to visfatin. These results suggested that visfatin can drive the neighboring ADSCs to stimulate malignant behaviors in breast cancer cells.

### 2.2. Growth Differentiation Factor 15 (GDF15) Plays a Crucial Role in Promoting Malignant Behaviors

**Figure 3:**
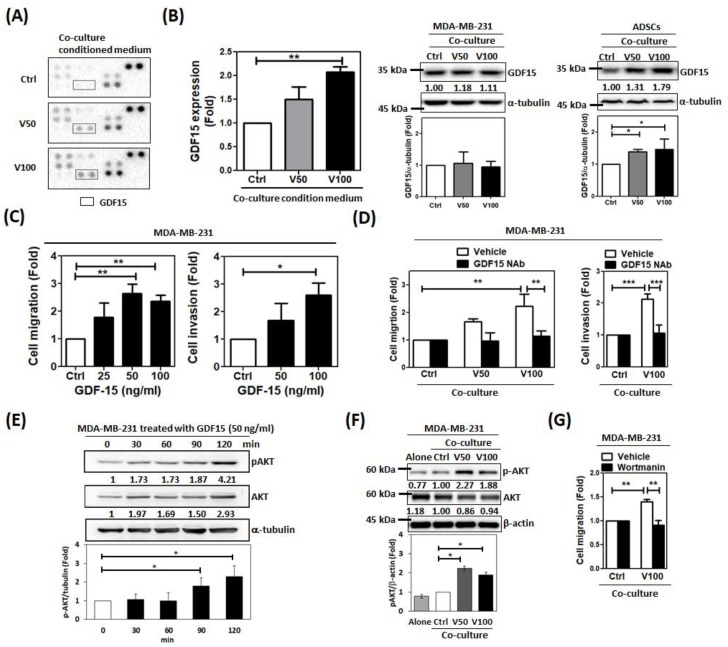
GDF15 play a crucial role in the regulations of breast cancer cells activities by visfatin-treated ADSCs (vADSCs). (**A**) After the three-day co-culture of MDA-MB-231 cells and vADSCs (V50 and V100 group) or untreated ADSCs (Ctrl group), the CM was collected and analyzed by using a cytokine array kit. (**B**) The expression of GDF15 in the co-cultured CM was validated by ELISA represented in a histogram. The ADSCs and MDA-MB-231 cells collected from the co-culture system were extracted for cell lysate to analyze the GDF15 expression by western blotting. (**C**) The migration and invasion of MDA-MB-231 treated with GDF15 at various concentrations for 48 h were evaluated by using a transwell system. (**D**) The indirect co-culture was performed in the presence or absence of the GDF15 neutralizing antibody of for three days. After that, the migration and invasion of the MDA-MB-231 cells collected from the co-culture were evaluated in a transwell system. (**E**) The expression of phosphor-AKT (pAKT) of MDA-MB-231 treated with GDF15 (50 ng/mL) at different time point was detected by western blotting. (**F**) The pAKT was detected in the MDA-MB-231 cells from the co-culture by western blotting. (**G**) After the three-day co-culture in the presence or absence of the wortmannin (400 nM), the MDA-MB-231 cells were collected from the co-culture for performing the migration assay. All experiments were performed in triplicate.

To investigate the mechanism of regulation, we used a cytokine array kit to analyze the condition medium (CM) of the co-culture. We found that the signal of growth differentiation factor 15 (GDF15) was stronger in the co-culture CM of vADSC and MDA-MB-231 than that of uADSCs and MDA-MB-231. This upregulation of GDF15 in the CM was confirmed by ELISA. GDF15 expression in the cells of uADSCs, vADSCs, and MDA-MB-231 collected from the co-culture system was examined by Western blotting. We found a slight increase in GDF15 expression in vADSCs but not in MDA-MB-231, which implied that GDF15 was produced by vADSCs (Figure 3B). The increased expression of GDF15 in ADSCs treated with visfatin was also confirmed by western blotting (Figure S3). Furthermore, we treated MDA-MB-231 cell with GDF15 recombinant proteins and found enhancement of migration and invasion of MDA-MB-231 cells (Figure 3C). To confirm the role of GDF15 in the CM, GDF15 neutralizing antibody was added to the co-culture of vADSC and MDA-MB-231. This reversed the enhancement of migration and invasion of MDA-MB-231 by co-culture (Figure 3D). Since GDF15 has been reported to activate the AKT pathway [28,29], we examined the expression of phosphor-AKT (pAKT) of MDA-MB-231 and found its induction within two hours after GDF15 treatment (Figure 3E). We checked the level of pAKT in MDA-MB-231 after co-culture and found it was higher in co-culture with vADSCs than with uADSCs (Figure 3F). By incubating with the PI3K/AKT inhibitor wortmannin in the co-culture system, the enhancement of MDA-MB-231 cell migration by ADSCs was inhibited (Figure 3G).

**Figure 4:**
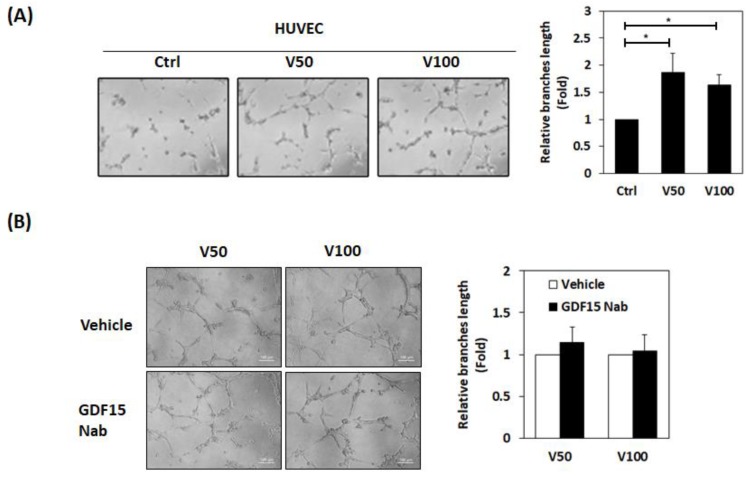
Visfatin-primed ADSCs promoted the tube formation of HUVEC. (**A**) HUVEC cells were co-cultured with visfatin-treated ADSCs or untreated ADSCs, noted as V50 and V100 or Ctrl, respectively, for three days. The HUVEC cells were collected from the co-culture and seeded in a matrix gel-coated 96-well plate. The tube formation of HUVEC was observed using a microscope. The length of branches was determined by using the ImageJ software. (**B**) After the three-day co-culture in the presence or absence of GDF15 neutralizing antibody (GDF15 Nab, 5 μg/mL), the HUVEC cells were collected from the co-culture for performing the tube formation assay. The experiments were performed in triplicate.

Angiogenesis was assessed with HUVEC tube formation assay, by co-culture of HUVECs with vADSCs or uADSCs. We found vADSCs stimulated tube formation of HUVECs better than uADSCs (Figure 4A), but this effect was not abolished by GDF15 neutralizing antibody in co-culture with vADSCs (Figure 4B). These findings suggested that the generation of GDF15 in co-culture of MDA-MB-231 and vADSCs play a key role in promoting MDA-MB-231 malignant behaviors via pAKT pathway, independent of its effects on angiogenesis.

### 2.3. Visfatin-Pretreated ADSCs Enhanced Breast Cancer Tumor Growth and Metastasis in a Xenograft Mouse Model

**Figure 5:**
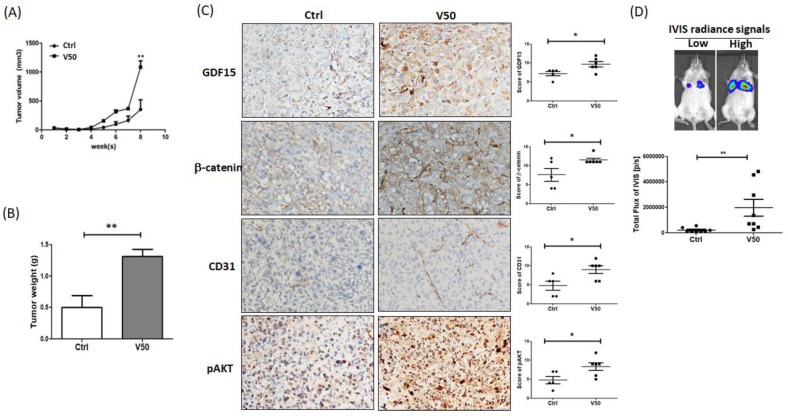
Visfatin-pretreated ADSCs (vADSCs) enhanced the tumor growth and metastasis in human breast cancer xenograft mouse model. (**A**) The nude mice were injected with mixture of MDA-MB-231 and untreated ADSCs (uADSCs) or vADSCs, noted as Ctrl or V50, respectively, to the mammary fat pads. The tumor volumes were measured every week after injection (Ctrl, n = 5; V50, n = 6). (**B**) After sacrificing the mice, the weight of the resected tumor was measured. (**C**) The expressions of GDF15, β-catenin, CD31, and pAKT in the tumor sections were detected by immunohistochemistry. The IHC score was calculated by multiplying the percentage of positive cells by the intensity and present as histogram. (**D**) The luciferase-expressing MDA-MB-231 were collected and injected into the tail vein of NOD/SCID mice after co-culturing with uADSCs or vADSCs, noted as Ctrl or V50, respectively (Ctrl, n = 8; V50, n = 8). The IVIS radiance signals of the mice were assessed at week 4. The representative images of high and low signal were shown. The statistical differences were calculated by t-test, *, *p*-value < 0.05; **, *p*-value < 0.01.

To explore the effect of visfatin-pretreated ADSCs (vADSCs) on MDA-MB-231 tumor formation in vivo, we used an orthotopic xenograft nude mouse model formed by co-injecting MDA-MB-231 and vADSCs or untreated ADSCs (uADSCs), noted as V50 or Ctrl, respectively, into the mammary fat pads subcutaneously. This result showed in significantly larger tumor formation with V50 versus Ctrl at week 8 (Figure 5A). After sacrificing the mice, the weight of V50 tumors was found to be significantly higher than that of Ctrl tumors (Figure 5B). The tumor tissues were collected to analyze related protein expressions by immunohistochemistry. Expression of GDF15, β-catenin, pAKT, and CD31 were significantly higher in V50 tumors than in Ctrl tumors (Figure 5C). Furthermore, the effect on tumor metastasis was examined by using mouse tail-vein injection model. The luciferase-expressing MDA-MB-231 cells were collected to inject into the tail vein of NOD/SCID mice after co-culturing with vADSCs or uADSCs for three days, noted as V50 or Ctrl, respectively. The result showed that co-cultured with vADSCs increased lung metastasis of the luciferase-expressing MDA-MB-231 cells more than co-cultured with uADSCs, determined by photo flux reading at week 4 (Figure 5D).

### 2.4. Correlation Analysis Between the Expression of Visfatin, GDF15, and pAKT in the Specimen of Breast Cancer Patients

**Figure 6:**
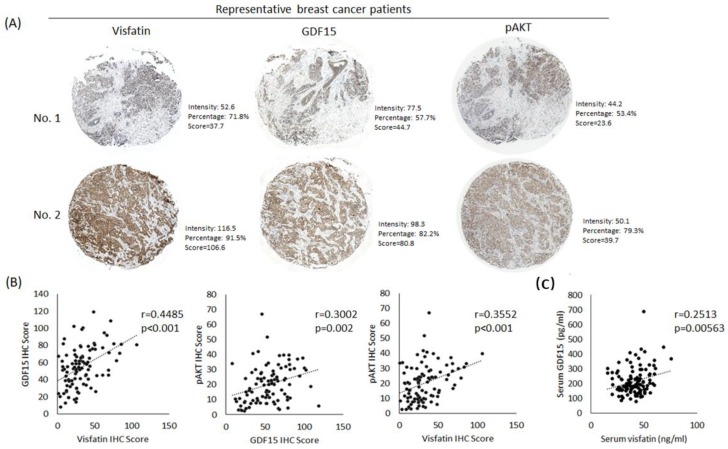
The expressions of visfatin, GDF15, and pAKT in the specimens from breast cancer patients. (**A**) The expressions of visfatin, GDF15, and pAKT in breast cancer tissue microarray (n = 96) were detected by immunohistochemistry. The representative images of high expression levels (No. 1) and low expression levels (No. 2) were shown. The IHC score was calculated by multiplying the percentage of positive cells by the intensity, which was identified using HistoQuest Analysis Software. (**B**) The correlations between visfatin, GDF15, and pAKT according to the IHC score were calculated by using the online Pearson correlation coefficient calculator. (**C**) The correlation of serum levels of GDF15 and visfatin of breast cancer patients (n = 120) determined by ELISA was also calculated by using the online Pearson correlation coefficient calculator.

To validate the correlation between the expression of visfatin, GDF15, and pAKT in breast cancer specimens, the immunohistochemistry of these three proteins was assayed in human breast cancer tissue microarray samples (Figure 6A). Staining results were captured and analyzed with TissueFAXS 3.5 and HistoQuest Analysis Software. Correlation was analyzed with the Pearson correlation coefficient (https://www.socscistatistics.com/tests/pearson/default2.aspx). The results showed that visfatin and GDF15 had a positive correlation (r = 0.4485, *p* < 0.001), GDF15 and pAKT had a positive correlation (r = 0.3002, *p* = 0.002), and visfatin and pAKT had a positive correlation (r = 0.3552, *p* < 0.001) (Figure 6B). Further, we examined the serum levels of visfatin and GDF15 of breast cancer patients by ELISA. We found that visfatin and GDF15 had a positive correlation (r = 0.2513, *p* = 0.005) in the peripheral blood of the breast cancer patients (Figure 6C). We also analyzed the Oncomine database and found the expression level of GDF15 transcript was significantly higher in invasive ductal breast carcinoma tissues than that in normal breast tissues (Figure S4). 

## 3. Discussion

### 3.1. Adipocytokines, ADSCs and the Tumor Microenvironment

**Figure 7:**
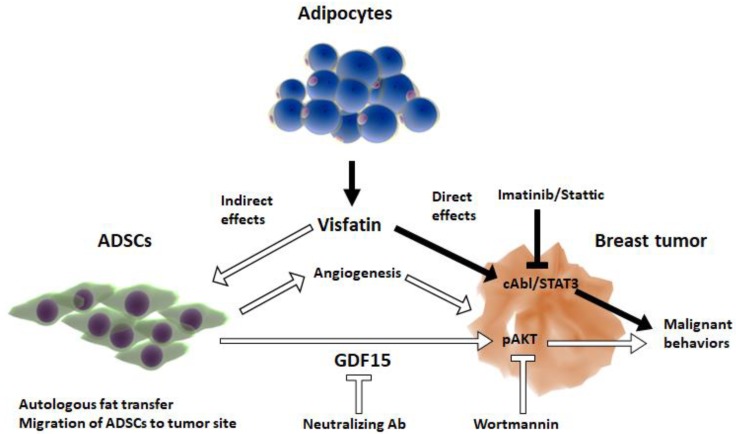
Visfatin mediates its effects both directly via cAbl/STAT3 and indirectly mediated by ADSCs via GDF15/AKT on promoting malignant behavior in breast cancer. Previously, we discovered visfatin mainly produced by adipocytes promoted breast cancer cells directly through activation of c-Abl and STAT3, which was blocked by Imatinib and Stattic inhibitor, respectively (black arrow). In this study, we showed that visfatin can act via an indirect pathway by priming ADSCs, which may be recruited from the adipose tissue to tumor site or generated from autologous fat transfer, to produce GDF15 that stimulated AKT activation in breast cancer cells to promote malignant behaviors (white arrow). The effect can be blocked by the treatment of GDF15 neutralizing Ab or Wortmannin inhibitor.

The data presented here add to a growing body of literature indicating that stromal-tumor interactions are of profound significance in breast cancer, and specifically this is the first study to use an adipocytokine-ADSCs-tumor cell line co-culture model. Here, we show that visfatin can act via mechanistically distinct pathways from those previously discovered using tumor cell line models in isolation [23], and that these newly discovered pathways are mediated via ADSCs in the tumor microenvironment (Figure 7). This may have significant future implications on the relevance of using tumor cell lines in isolation in breast cancer research. Furthermore, this study also suggests a re-evaluation of factors that may affect ADSCs in the tumor micro-environment, including hormonal therapy, radiotherapy, and autologous fat grafting in breast cancer and obesity. 

Obesity may influence breast cancer progression through alteration of systemic metabolism, inflammatory response, growth factor signaling, and angiogenesis. A key component in this process is ADSCs, which are present in breast tissue at approximately 0.6 × 10^6^ ADSCs per gram of tissue [30]. During obesity progression, adipose tissue expansion leads to adipocytokine overproduction and ADSC proliferation. The increased ADSCs may traffic from the adipose tissue to tumor to accelerate cancer progression [31,32,33]. A mouse model of diet-induced obese (DIO) has suggested that obesity-induced secretion of CXCL1 by cancer cells creates a chemotactic gradient that enables ADSCs trafficking to tumors via CXCR1 [34]. The inflammatory cytokines MIP-1δ/MIP-3α [35], and PDGF BB/PDGR-B have also been implicated in ADSCs tumor tropism [36], with PDGF BB expression levels elevated after radiotherapy treatment. Furthermore, systemic migration of ADSCs to tumor sites has been demonstrated with fluorescence labelled ADSCs tail vein injection mouse models [35]. In the present study, we found that visfatin-treated ADSCs promoted malignant behaviors and tumor formation in breast cancer cells via a GDF15-induced AKT pathway. Previous data has demonstrated that co-injection of ADSCs with MDA-MB-231 stimulated greater tumor volume in mammary fat pad of nude mice than MDA-MB-231 injection alone [15]. Here we co-cultured MDA-MB-231 with ADSCs in a transwell, and co-injected MDA-MB-231 with ADSCs into a mouse mammary fat pad to mimic breast tumor recruitment of ADSCs. ADSCs pretreated with visfatin had a more significant effect on MDA-MB-231 tumor formation than untreated ADSCs, implying that ADSCs immersed in a visfatin-rich environment of obese adipose tissue may enhance its promoting effect on breast cancer progression.

### 3.2. Visfatin-Primed ADSCs Promote Tumor Stemness and EMT through GDF15-pAKT Pathway 

Exogenous administration of visfatin can stimulate human leukocytes to produce IL-1β, TNF-α, and IL-6 [37], or induce the secretion of IL-6, IL-8, and MCP-1 during osteogenic and adipogenic differentiation of MSCs [38]. Visfatin may also activate HUVEC to up-regulate the expression of VEGF and MMPs [39]. In the present study, we found that co-culture of CM of MDA-MB-231 and vADSCs increased GDF15 expression, which was vADSC specific. GDF15 (also known as MIC-1, NAG-1, PLAB, PTGFB) is a cytokine of the TGF-β superfamily that is up-regulated in response to inflammation, cardiovascular disease, obesity, and cancer [40,41,42]. GDF15 may be involved in the proliferation, migration, invasion, and angiogenesis of tumors, with with recent research finding GDF15 promoted EMT and invasion of breast cancers [43], and supported the maintenance of breast cancer stem-like cells [44]. Furthermore, GDF15 promoted the proliferation of cervical cancer cells by interaction with ErB2 to activate PI3K/AKT and MAPK/ERK pathways [28], and enhanced the migration of pancreatic cancer cells via an AKT pathway [29]. Similarly, our study found that increased pAKT levels in MDA-MB-231 co-culture were responsible for tumor cell migration-promoting effects. This was reversed with GDF15 neutralizing antibody and wortmannin inhibitor. Although GDF15 has been reported by others to promote angiogenesis in tube formation assays with HUVEC cells [45], we found that treatment with GDF15 neutralizing antibody did not inhibit the angiogenic effect from vADSCs. This implied that a GDF15 independent pathway may be involved in vADSCs related angiogenesis. Although this was not the focus of our study, mechanisms described for ADSCs related angiogenesis include elevated VEGF production leading to angiogenic sprouting, pericyte differentiation, and the IL-6 endothelin 1 pathway [46,47]. Collectively our findings suggested that vADSCs may mediate breast cancer EMT and stemness via a GDF15-induced AKT pathway, and promote angiogenesis through a GDF15 independent pathway.

GDF15 protein expression is markedly increased in various types of cancer biopsies including breast cancer [43,48,49,50,51], with our analysis of the Oncomine database also noting significantly higher GDF15 mRNA expression levels in breast cancer tissues than normal breast tissues. In this study, we also showed a positive correlation between GDF15 and visfatin expression in breast tumors. As an association between visfatin and breast cancer progression has been previously established [22,23,52], GDF15 may likewise be used as a potential biomarker in breast cancer. Recently, the GDNF-family receptor α-like (GFRAL) protein was identified as a receptor for GDF15, with increased pAKT elicited by GDF15 in GFRAL-overexpressed HEK293 cell lines [53,54,55]. GFRAL has been proposed as a drug target for appetite-related disorders, and similarly may provide a future therapeutic strategy in breast cancer. In this regard, GFRAL may act as both an upstream and downstream therapeutic target, with appetite suppression reducing both obesity and correlated visfatin levels, and GDF15 inhibition reducing downstream visfatin mediated pAKT. An obese mouse-orthotopic breast cancer cell line model would act as an in-vivo analogue, allowing differential analysis of these effects.

### 3.3. Visfatin-ADSC-Tumor Co-Culture Is a Model for Future Breast Tumor Microenvironment Research

Currently, a significant proportion of breast cancer research is carried out on breast cancer cell lines that are not representative of the breast tumor microenvironment and cellular diversity, with some common cell lines derived from malignant pleural effusions. Weigand et al and others [56], suggest that only an ensemble of tissue including ADSCs, mammary epithelial cells and mesenychmal cells, can represent breast tumor tissue adequately. Our study is the first adipocytokine-ADSC-cancer line co-culture model in breast cancer, and the first to demonstrate that visfatin can act via mechanistically distinct pathways from those previously found using tumor cell line models in isolation [23]. Therefore, basing breast cancer research on tumor cell lineage models alone, may result in incomplete elucidation of tumor pathways with a potentially reduced efficacy of translational clinical therapies. 

## 4. Materials and Methods

### 4.1. Cell Culture

The human breast carcinoma cell lines MCF7 and MDA-MB-231, and human umbilical vein endothelial cell line HUVEC were purchased from the Bioresource Collection and Research Center (BCRC, Hsinchu, Taiwan). The luciferase-expressing MDA-MB-231 cells were kindly provided by Prof. Wen-Chun Hung, National Health Research Institutes, Taiwan. MCF-7, MDA-MB-231, and luciferase-expressing MDA-MB-231 cells were cultured in DMEM medium (12100046, Thermo Fisher Scientific, Waltham, MA, USA) supplemented with 10% FBS (Biological Industries, Cromwell, CT, USA) and 1% antibiotic-antimycotic (15240062, Thermo Fisher Scientific). HUVEC was cultured in Media 199 (11150059, Thermo Fisher Scientific) with 10% FBS (Thermo Fisher Scientific), 30ug/ml endothelial cell growth supplement (ECGS) (E2759, Sigma-Aldrich, St Louis, MO, USA), and 1% antibiotic-antimycotic (15240062, Thermo Fisher Scientific). Human ADSCs isolation was modified based on previous article [57]. In brief, the ADSCs were isolated from tumor-free adipose tissue adjacent to the breast tumor of 10 breast cancer patients with invasive ductal carcinoma that underwent mastectomy. The patients signed informed consent document approved by the Institutional Review Board of Kaohsiung Medical University Hospital. The adipose tissues were cut into small pieces using scissors, and the extracellular matrix digested with 5 mg/mL collagenase type II (C6885, Sigma-Aldrich) at 37 °C for 1 h with gentle shaking. The cell solution was passed through 100 μm cell strainer (Falcon 352360, Corning, NY, USA). The filtrate was centrifuged at 500 g for 10 min. The cell pellet was washed with PBS and cultured with alpha minimum essential medium (α-MEM) (Hyclone SH30526.02, GE Healthcare, Pittsburgh, PA, USA) containing 5% UltraGRO (HPCPLCRL10, AventaCell, Atlanta, GA, USA). The total number of passages for experiment was up to seven passages. All cells were cultured with 5% CO2 at 37 °C in a humidified incubator.

### 4.2. ADSCs Differentiation

The differentiation of ADSCs into three cell lineages was performed according to a previous protocol with modification. For adipogenic differentiation, ADSCs were cultured in adipogenic medium (DMEM supplemented with 10% FBS, 100 µM indomethacin (I7378, Sigma-Aldrich), 10 µg/mL insulin (I3536, Sigma-Aldrich), 1 μM dexamethasone (D4902, Sigma-Aldrich), 0.5 mM isobutylmethylxanthine (IBMX) (I5879, Sigma-Aldrich), 100 U/mL penicillin, and 100 mg/mL streptomycin) for 12 days. Then, the cells were stained with Oil Red-O (O0625, Sigma-Aldrich) to examine the lipid accumulation. For osteogenic differentiation, ADSCs were cultured with osteogenic medium (DMEM supplemented with 10% FBS 10 mM β-glycerophosphate (G9422, Sigma-Aldrich), 0.05 mM ascorbate-2-phosphate (A8960, Sigma-Aldrich), 100 nM dexamethasone, 100 U/mL penicillin, and 100 mg/mL streptomycin) for 2 weeks. After that, the cells were stained with von Kossa (1003620001, Sigma-Aldrich) for evaluation. For chondrogenic differentiation, ADSCs were cultured with chondrogenic medium (DMEM supplemented with 10% FBS, 6.25 µg/mL insulin, 10 ng/mL TGF-β1 (100-21, Peprotech, Rocky Hill, NJ, USA), and 50 μM L-ascorbic-acid-2-phosphate (A8960, Sigma-Aldrich)) for 2 weeks. Then, the cells were stained with Alcian blue (A5268, Sigma-Aldrich) to examine the sulfated proteoglycan-rich matrix. All the culture medium was replaced every 2 or 3 days throughout the period of differentiation.

### 4.3. Flow Cytometry

For characterizing the ADSCs, the cells were collected and incubated with fluorochrome-conjugated antibodies against CD44 (11-0441-82, Thermo Fisher Scientific), CD90 (11-0909-42, Thermo Fisher Scientific), CD34 (12-0349-42, Thermo Fisher Scientific), CD45 (11-0459-42, Thermo Fisher Scientific), and CD105 (12-1057-42, Thermo Fisher Scientific). The stained cells were washed with PBS and suspended in sorting buffer before analysis by FC500 Flow Cytometer (Beckman Coulter, Brea, CA, USA).

### 4.4. Indirect Co-Culture 

After 3–6 passages, ADSCs were seeded in 6-well plate (2 × 10^5^ cells/well) overnight and then underwent serum starvation for 24 h. The ADSCs were treated with or without visfatin, noted as vADSCs or uADSCs, respectively, at 50 or 100 ng/mL, which mimic the physiological level of breast cancer patients [23], in α-MEM containing 0.5% UltraGRO for 48 h. After that, the indirect co-culture in a transwell was performed. The vADSCs or uADSCs were collected to seed in the inserts (2 × 10^4^ cells/insert) of 24-well transwell plate (0.4 μm pores, Corning) with 0.5% UltraGRO α-MEM. The MDA-MB-231 or HUVEC cells were seeded in the lower wells (6 × 10^4^ cells/well) with 1% FBS DMEM or 1% FBS Media 199, respectively. For testing the regulation mechanism, GDF15 neutralizing antibody (5 μg/mL) (MAB957-100, R&D systems, Minneapolis, MN, USA) or the PI3K/AKT inhibitor wortmannin (400 nM) (W1628, Sigma-Aldrich) were added in the lower well. After 72-hour co-culture, the MDA-MB-231 cells were collected for performing cell proliferation assay, soft agar colony formation assay, migration, invasion, and tumorsphere formation assays. The HUVEC cells were collected for tube formation assay.

### 4.5. Cell Proliferation Assay

The treated MDA-MB-231 cells were seeded in 96-well plate (2000 cells/well) and cultured with DMEM containing with 10% FBS for 24~72 h. After that, XTT (2,3-Bis-(2-methoxy-4-nitro-5-sulfophenyl)-2H-tetrazolium-5-carboxanilide, X4251, Sigma-Aldrich) was applied into wells with PMS (Phenazine methosulfate, P9625, Sigma-Aldrich). The absorbance (A1) of each well was measured at a wavelength of 475 nm and the nonspecific reading (A2) was measured at 660 nm. The proliferation rates were calculated as ΔA = A1 − A2.

### 4.6. Soft Agar Colony Formation Assay

A 6-well culture plates containing 0.5% agarose as solid bases were prepared. The treated MDA-MB-231 cells suspended with 10% FBS DMEM in 0.35% agarose was added to top of the solid base. The cells were cultured for 14 days to form colonies. Colonies were stained with 0.25% crystal violet, and the colonies with diameter above 1 mm were analyzed using a stereoscope dissecting microscope. The amounts of colonies were analyzed using ImageJ software (National Institute of Mental Health, Bethesda, MD, USA). 

### 4.7. Cell Migration and Invasion Assays

For performing cell invasion or migration assay, the MDA-MB-231 cells collected from the co-culture system or treated with human recombinant GDF15 (10936-H01H, Sino Biological, Wayne, PA, USA) were suspended in serum-free DMEM and transferred to the 24-well transwell inserts coated with or without Matrigel, respectively (8 μm pores, Corning). The lower wells were added with DMEM containing with 10% FBS. After 24-hour incubation, cells in the top chamber were removed by cotton swabs. The membrane-trapped cells were fixed, stained with crystal violet and observed using a light microscope. The amounts of migrated cells were analyzed using ImageJ software. 

### 4.8. Tumorsphere Formation Assay

After the indirect co-culture, MDA-MB-231 or MCF7 were collected to suspended in the phenol red-free DMEM (21063029, Thermo Fisher Scientific) supplemented with recombinant human fibroblast growth factor basic (20 ng/ml) (100-18C, Peprotech), recombinant human epidermal growth factor (20 ng/mL) (GMP100-15, Peprotech), insulin (10 μg/mL), and 1 × B27 (17504044, Thermo Fisher Scientific). The cells were seeded into an ultra-low attachment 96-well plate (3474, Corning) at a density of 2000 cells per well and cultured in a humidified incubator with 5% CO2 at 37 °C for 7 days. The amount of tumorspheres with diameter over 50 μm was counted.

### 4.9. Tube Formation Assay

Human umbilical cord vein endothelial cells (HUVECs) were treated with the CM of visfatin-treated ADSCs or indirectly co-cultured with visfatin-treated ADSCs as describe above. Meanwhile, a 96-well containing 100 μL Matrigel per well (354234, Corning) was first chilled at 4 °C overnight. Then, the Matrigel-containing plate was incubated at 37 °C, 5% CO2 for 30 min. After that, the treated HUVECs were seeded on the Matrigel (1 × 10^4^ cells/well) and incubated for 4 h. The tube formation was observed by a light microscope and the network length (cells joined from end-end) was analyzed by ImageJ software. 

### 4.10. Western Blot

The ADSCs and MDA-MB-231 cells collected from the co-culture system or GDF15-treated MDA-MB-231 cells were lysed with RIPA lysis buffer. Protein concentrations determined using Bio-Rad protein assay (BIO-RAD, Hercules, CA, USA). Equal amounts of total proteins were subjected to sodium dodecyl sulfate–polyacrylamide gel electrophoresis, and transferred to nitrocellulose membrane (IBFP0785C, Merck Millipore, Burlington, MA, USA). After blocking with 5% non-fat milk, the membrane was incubated with primary antibodies at 4 °C overnight. Primary antibodies used in western blotting included β-actin (GTX109639, GeneTex, Hsinchu, Taiwan), α-tubulin (GTX112141), ZEB1 (GTX105278), Snail (GTX82509), ZO-1 (GTX108627), Nanog (GTX100863), Oct4 (GTX101497), β-catenin (ab16051, Abcam, Cambridge, MA, USA), GDF15 (PAB31426, Abnova, Taipei, Taiwan), phosphor-AKT (4060, Cell Signaling, Danvers, MA, USA), and AKT (4691, Cell Signaling). Immunoreactive proteins were detected after incubation with horseradish peroxidase-conjugated secondary antibody (31430, Thermo Scientific) for 1 h at room temperature. The immunoblots were visualized using chemiluminescence reagent (WBKLS0500, Merck Millipore) and quantified by ChemiDoc XRS+ imaging system (BIO-RAD).

### 4.11. Cytokine Array Analysis and ELISA

To explore the cytokine expression profile in the medium of the indirect co-culture of MDA-MB-231 and resistin-pretreated ADSCs, a Proteome profiler™ human XL cytokine array kit (ARY022, R&D Systems) was adopted. The expressions of GDF15 in the co-cultured medium and the serum of breast cancer patients obtained at the Cancer Center of Kaohsiung Medical University Hospital (KMUH) during the 2003 to 2008 from previous study [22] were detected by using a human GDF15 ELISA kit (DGD150, R&D Systems). The visfatin expression in the serum of breast cancer patients were detected by using a human visfatin ELISA kit (DY4335-05, R&D Systems). These kits were used according to manufacturer’s instructions.

### 4.12. Animal Study

The animal experiments were conducted in accordance with the Institutional Animal Care and Utilization Committee of Kaohsiung Medical University, Kaohsiung, Taiwan. The number of animals used in the experiments was minimized according to the 3Rs. Nude mice (BALB/cAnN.Cg-Foxn1nu/CrlNarl) aged 6–8 weeks were purchased from the National Laboratory Animal Center, Taiwan. The nude mice were fed with normal diet under specific pathogen-free conditions. For orthotopic model, 1 × 10^6^ visfatin-treated ADSCs (vADSCs) or untreated ADSCs (uADSCs) were co-injected with 2 × 10^6^ MDA-MB-231 cells into the 4th mammary fat pads of female nude mice. The size of tumor was measured twice a week, and tumor volumes calculated according to the standard formula: (Width 2 × Length)/2. After 45 to 60 days, the mice were sacrificed and tumors, organs were collected for evaluation. For metastatic model, the luciferase-expressing MDA-MB-231 cells were co-cultured with vADSCs or uADSCs for 3 days. After that, 2 × 10^5^ luciferase-expressing MDA-MB-231 cells were collected to inject into the tail vein of NOD/SCID mice (NOD.CB17-Prkdcscid/NcrCrl; aged 6-8 weeks; National Laboratory Animal Center, Taiwan). At week 4, the mice were injected with luciferin and the bioluminescent signal was assessed using an IVIS50 in vivo imaging system (Xenogen, Alameda, CA, USA). The total flux of IVIS radiance signal was calculated. 

### 4.13. Immunohistochemistry

The MDA-MB-231 tumors of the orthotopic xenograft mouse model were collected to make formalin-fixed paraffin-embedded tissue blocks. The tissue microarray slides of human breast cancer specimens obtained at the Cancer Center of Kaohsiung Medical University Hospital (KMUH) from 2003 to 2008 were generated by previous study [22]. The immunohistochemistry was performed on 5 μm paraffin sections by using a fully automated Bond-Max System (Leica Microsystems, Wetzlar, Germany). All staining steps were performed by the automated instrument, according to the manufacturer’s instructions (Leica Microsystems). The primary antibodies used for this experiment included visfatin (sc-376336, Santa Cruz, Dallas, Texas), GDF15 (PAB31426), β-catenin (ab16051), and phosphor-AKT (GTX28932). The staining of the xenograft tumors was determined separately for each specimen by 2 independent experts simultaneously under the same condition. The staining result was calculated by multiplying the score of percentage of positive cells by the score of staining intensity. The score of percentage of positive cells was defined as: score 0, 0%; score 1, 1%~25% positive cells; score 2, 26%~50%; score 3, 51%~75%; score 4, 76%~100%. The score of staining intensity was defined as: score 0, negative; score 1, weak; score 2, moderate; score 3, strong. The staining of the human breast cancer tissue microarray slides was scanned by using TissueFAXS 3.5 (TissueGnostics, Vienna, Austria). The percentage of positive cells and the staining intensity were analyzed by using HistoQuest Analysis Software (TissueGnostics). The staining result was also calculated by multiplying the score of percentage of positive cells by the score of staining intensity. 

### 4.14. Statistical Analysis

Statistical analysis was performed using the GraphPad Prism 5 software (GraphPad Software Incorporation, San Diego, CA, USA). All the data were shown as mean ± SEM. Student’s t-test was used to compare two groups. Significance differences were set as *p < 0.05; ** *p* < 0.01; *** *p* < 0.001. The correlation of proteins expression detected by IHC or ELISA was calculated by using the online Pearson correlation coefficient calculator (https://www.socscistatistics.com/tests/pearson/default2.aspx).

## 5. Conclusions

The data presented here support the accumulating evidence supporting the importance of stromal-tumor interactions in breast cancer. Significantly, this is the first study to use an adipocytokine-ADSCs-tumor cell line co-culture model. Here, we showed that visfatin can act both directly on tumor cells and indirectly via ADSCs in the tumor microenvironment. The indirect pathway, mediated by visfatin-primed ADSCs, promoted tumor stemness and EMT through a GDF15-pAKT pathway. This study establishes a novel and important model for future stromal-tumor analysis in breast cancer, and highlights previously unknown therapeutic targets in the GDF15-pAKT pathway.

## Supplementary Materials

The following are available online at https://www.mdpi.com/2072-6694/12/1/29/s1, Figure S1: The effect of ADSCs treated with or without visfatin on the cell migration of MDA-MB-231, Figure S2: The cell migration of MDA-MB-231 co-cultured with visfatin-primed or unprimed ADSCs isolated from non-cancer patients underwent cosmetic breast surgery, Figure S3: The expression of GFD15 in ADSCs treated with or without visfatin, Figure S4: Analysis of the expression levels of GDF15 transcripts in Oncomine database.
