# Supplementary material for: Visfatin Mediates Malignant Behaviors through Adipose-Derived Stem Cells Intermediary in Breast Cancer
Source: Cancers (Basel). 2019 Dec 20;12(1):29. doi: 10.3390/cancers12010029 (PMC7016886; doi:10.3390/cancers12010029)
Supplement: Supplementary file 1 [file cancers-12-00029-s001.zip › cancers-633319-supplementary materials.pdf]

cancers

Supplementary Materials:

Visfatin Mediates Malignant Behaviors through Adipose-Derived Stem Cells Intermediary in Breast Cancer

Jyun-Yuan Huang, Yen-Yun Wang, Steven Lo, Ling-Ming Tseng, Dar-Ren Chen, Yi-Chia Wu, Ming-Feng Hou and Shyng-Shiou F. Yuan *

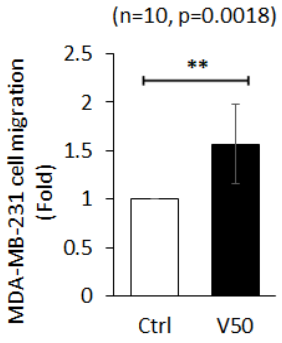

Figure S1. The effect of ADSCs treated with or without visfatin on the cell migration of MDA-MB-231. ADSCs were isolated from 10 breast cancer patients. The ADSCs were treated with or without visfatin (50 ng/ml), noted as V50 or Ctrl, respectively, for 48 h and then co-cultured with MDA-MB-231 for 72 h. After that, the MDA-MB-231 cells were collected to perform cell migration in a transwell system. The comparison of the cell migration between V50 and Ctrl groups was shown in histogram. The statistical differences were calculated by t-test, **, p-value < 0.01.

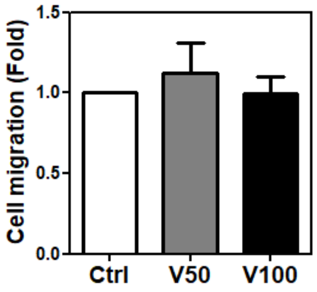

Figure S2. The cell migration of MDA-MB-231 co-cultured with visfatin-primed or unprimed ADSCs isolated from non-cancer patients underwent cosmetic breast surgery. ADSCs were isolated from 3 non-cancer patients underwent cosmetic breast surgery. The non-tumor ADSCs were treated with visfatin (50 ng/ml and 100 ng/ml), noted as V50 and V100, or without visfatin, noted as Ctrl, for 48 h and then co-cultured with MDA-MB-231 for 72 h. After that, the MDA-MB-231 cells were collected to perform cell migration in a transwell system.

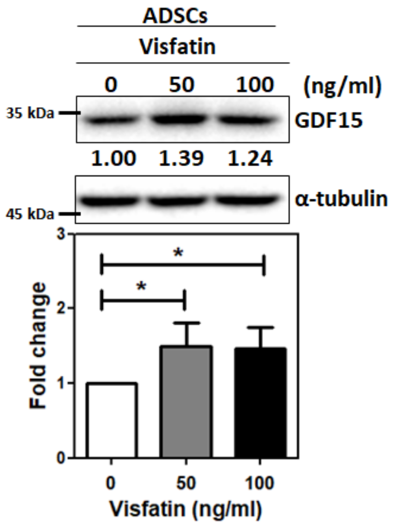

Figure S3. The expression of GFD15 in ADSCs treated with or without visfatin. The ADSCs with 3-6 passages were treated with visfatin at 50 or 100 ng/ml for 48 hours. Untreated ADSCs were served as control. Then, the expression of GDF-15 in the cells was determined by western blotting. The experiment was performed in triplicate.

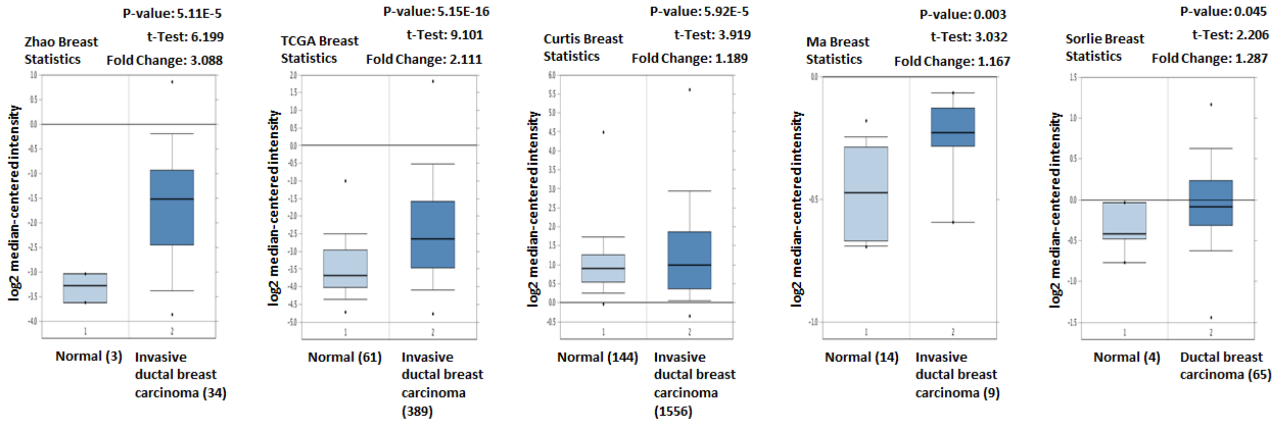

Figure S4. Analysis of the expression levels of GDF15 transcripts in Oncomine database. The GDF15 expression of invasive ductal breast carcinoma was higher than that of normal breast according to three different data base (https: // www. oncomine. org/ resource/ login.html).
