# Supplementary material for: Visfatin Mediates Malignant Behaviors through Adipose-Derived Stem Cells Intermediary in Breast Cancer
Source: Cancers (Basel). 2019 Dec 20;12(1):29. doi: 10.3390/cancers12010029 (PMC7016886; doi:10.3390/cancers12010029)

Supplementary figures

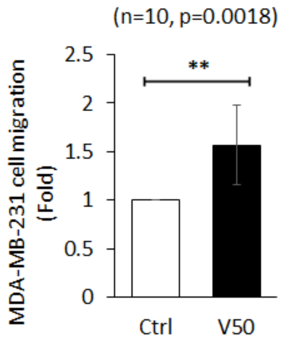

Supplementary Figure 1. The effect of ADSCs treated with or without visfatin on the cell migration of MDA-MB-231. ADSCs were isolated from 10 breast cancer patients. The ADSCs were treated with or without visfatin (50 ng/ml), noted as V50 or Ctrl, respectively, for 48 h and then co-cultured with MDA-MB-231 for 72 h. After that, the MDA-MB-231 cells were collected to perform cell migration in a transwell system. The comparison of the cell migration between V50 and Ctrl groups was shown in histogram. The statistical differences were calculated by t-test, **, p-value < 0.01.

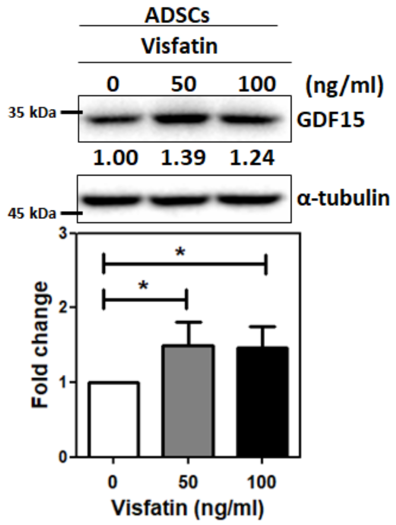

Supplementary figure 2. The expression of GFD15 in ADSCs treated with or without visfatin. The ADSCs with 3-6 passages were treated with visfatin at 50 or 100 ng/ml for 48 hours. Untreated ADSCs were served as control. Then, the expression of GDF-15 in the cells was determined by western blotting. The experiment was performed in triplicate.

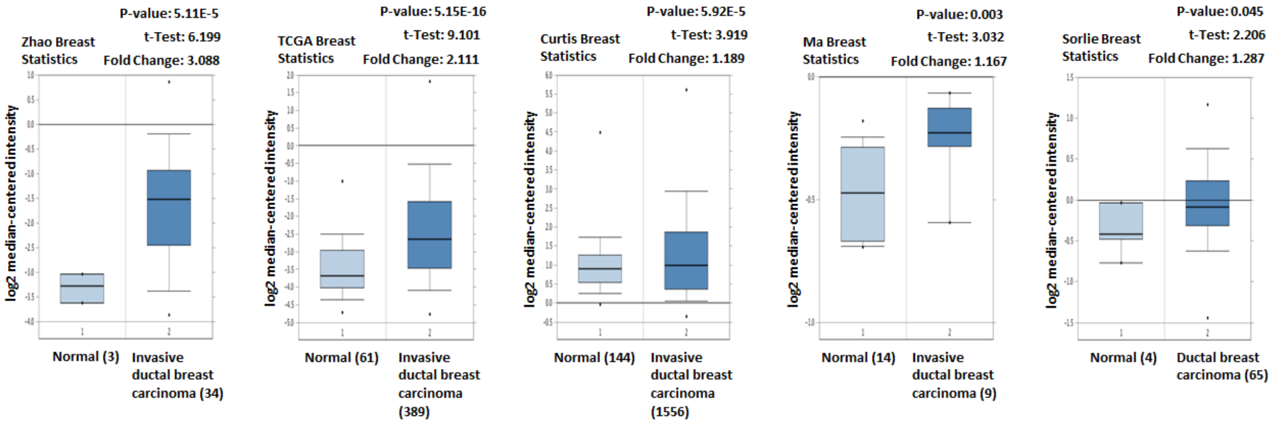

Supplementary figure 3. Analysis of the expression levels of GDF15 transcripts in Oncomine database. The GDF15 expression of invasive ductal breast carcinoma was higher than that of normal breast according to three different data base (https: // www. oncomine. org/ resource/ login.html).

Supplementary materials

Figure 2 (E) The collected MDA-MB-231 cells were analyzed for the expressions of EMT- and stemness-related proteins by western blotting. MDA-MB-231 cells only were noted as Alone.

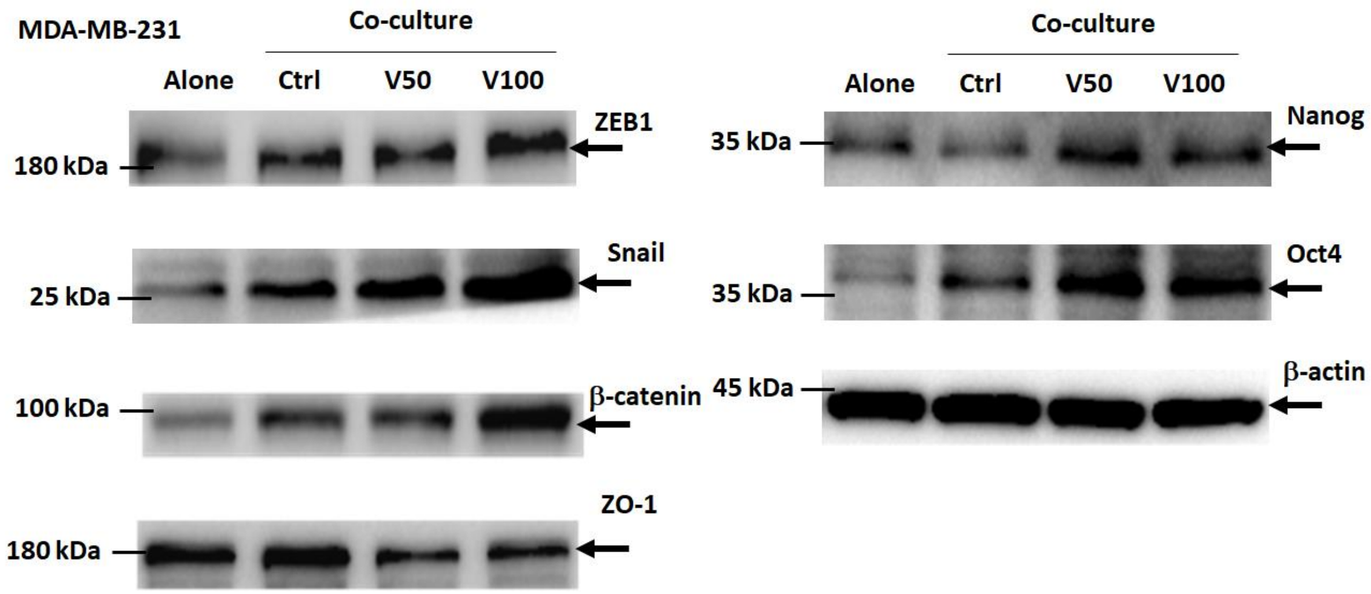

Figure 3 (B) The ADSCs and MDA-MB-231 cells collected from the co-culture system were extracted for cell lysate to analyze the GDF15 expression by western blotting.

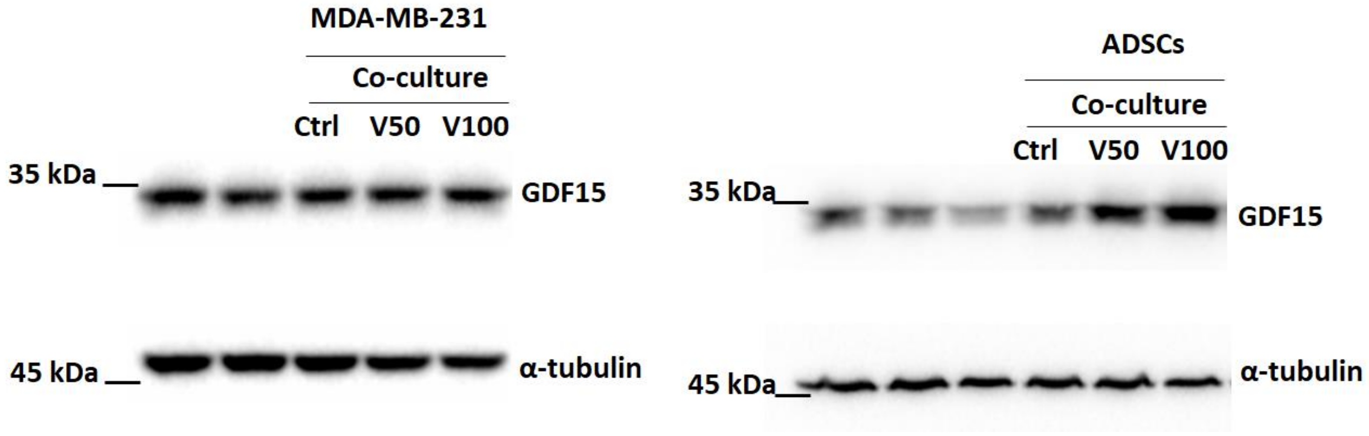

Figure 3 (E) The expression of phosphor-AKT (pAKT) of MDA-MB-231 treated with GDF15 (50 ng/ml) at different time point was detected by western blotting.

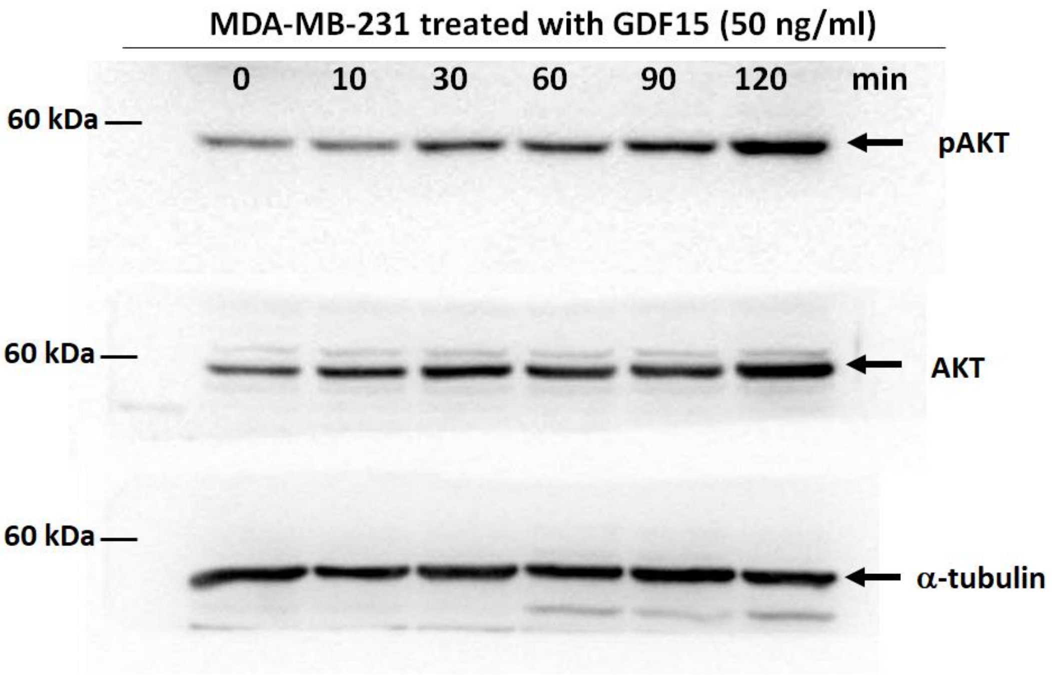

Figure 3 (F) The pAKT was detected in the MDA-MB-231 cells from the co-culture by western blotting.

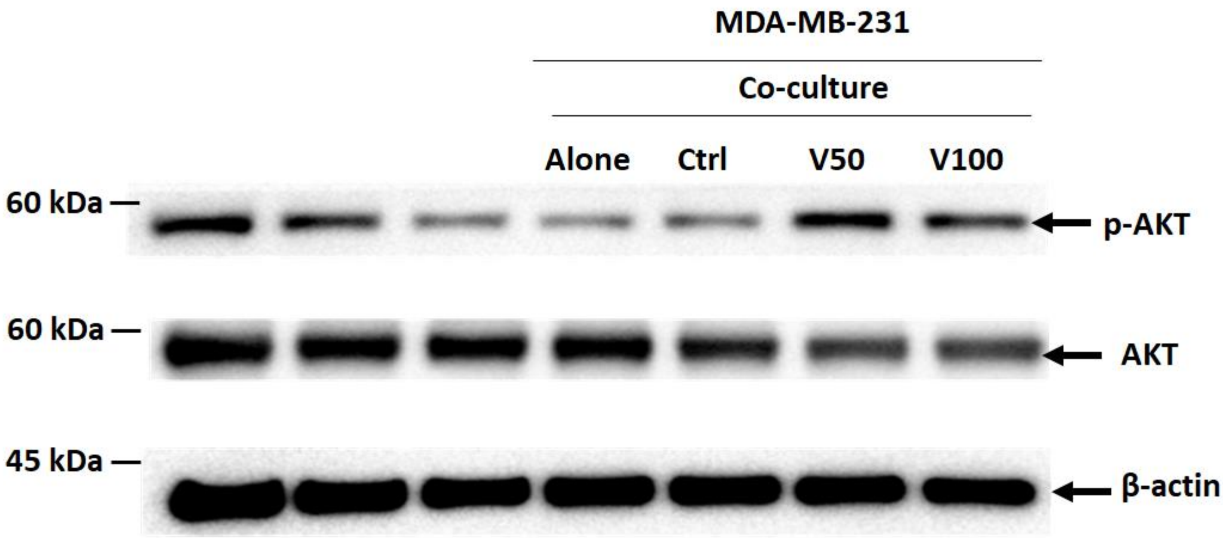

Supplementary Figure 2. The expression of GFD15 in ADSCs treated with or without visfatin. The ADSCs with 3-6 passages were treated with visfatin at 50 or 100 ng/ml for 48 hours. Untreated ADSCs were served as control. Then, the expression of GDF-15 in the cells was determined by western blotting.

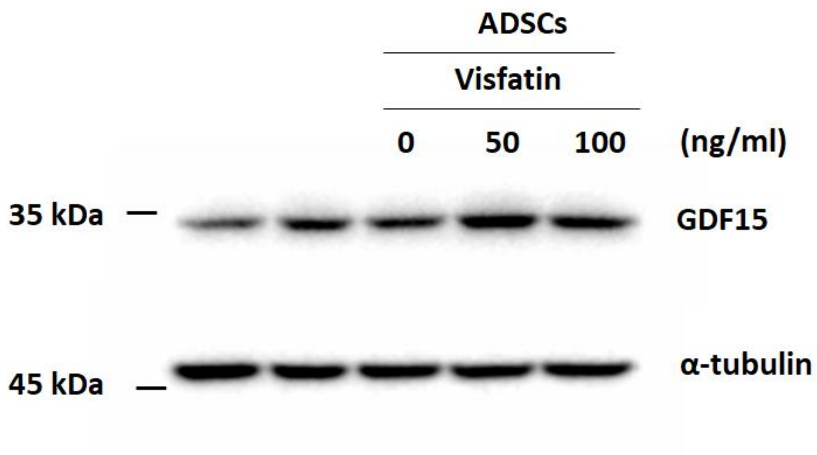

Supplement: Supplementary file 1 [file cancers-12-00029-s001.zip › cancers-633319-western blot.pdf]
